# Practices, Attitudes, and Knowledge Regarding Recombinant Zoster Vaccine Among Family Medicine Residents in Riyadh, Saudi Arabia

**DOI:** 10.7759/cureus.66301

**Published:** 2024-08-06

**Authors:** Raed Alahmari, Osama Alamri, Abdullah I Altashlan, Abdulaziz A Alsheikh, Mohammed Aljaloud

**Affiliations:** 1 Family Medicine, King Fahad Medical City, Riyadh, SAU; 2 Family Medicine, Ministry of Health (MOH) 2nd Health Cluster, Riyadh, SAU

**Keywords:** immunization education, knowledge gaps, vaccine availability, healthcare provider attitudes, vaccination recommendations, public awareness about vaccination, varicella-zoster, family medicine residency, kingdom of saudi arabia (ksa), varicella vaccines

## Abstract

Background

The aim of the research is to determine the existing knowledge, perceived practices, and attitudes toward the recombinant Zoster vaccine among family medicine residents (FMR) included in the medical profession. The present study aims to narrow down the identified gap in knowledge and develop vaccinations that will assist the targeted deme to eradicate zoster and the aftermaths that accompany it.

Methods

This research utilizes a descriptive cross-sectional survey design to assess the knowledge, practices, and attitudes of FMR toward the zoster vaccine in Riyadh, Saudi Arabia. By quantifying data at a specific point in time, this design allows for a detailed examination of the current status across various levels of residency programs. Participants from different institutions are interviewed simultaneously, enabling a thorough study of the targeted population group.

The study includes 154 FMR from three different levels (R1, R2, R3) enrolled in residency programs at various institutions in Riyadh, Saudi Arabia. These participants were selected from a group of individuals invited to share their prior knowledge, habits, and beliefs regarding the recombinant Zoster vaccine.

The study offers detailed statistical insights into demographics, vaccination attitudes, and knowledge among healthcare professionals. Key findings highlight diverse recommendations for different adult groups, the prevalence of vaccine availability, and the main sources of immunization information.

Results

The study found diverse recommendations for vaccination among different adult groups, with mean recommendations ranging from 2.50 to 2.94. Nearly all respondents (96.8%) reported having the vaccine available at their place of practice. However, knowledge gaps were evident, particularly concerning vaccination timing and specific requirements, highlighting the need for targeted education and clearer guidelines in vaccination practices among healthcare providers.

Conclusion

The study highlights the nuanced vaccination recommendations among healthcare professionals, particularly for different adult populations, and the availability of varicella-zoster virus (VZV) vaccines. The reliance on diverse information sources underscores the need for targeted educational efforts to ensure accurate and consistent immunization practices across healthcare settings. Addressing uncertainties and promoting informed decision-making can enhance vaccination uptake and patient care outcomes in clinical practice.

## Introduction

Shingles, commonly called herpes zoster (HZ), is a skin rash that is very painful and occurs when the chickenpox virus (varicella-zoster virus (VZV)) that has been inactive for a long time reactivates in people who already have had chickenpox [1]. The most common presentation is a rash on the trunk within a dermatome supplied by the thoracic nerve. In some cases, the rash may be less common and may involve three or more dermatomes which are separate and distinct areas of the body. This zoster constitutes a disseminated form of this virus. This typically only occurs in individuals who have a weakened or suppressed immune system. Zoster disseminated are hard to detach from varicella. The rash is often accompanied by pain, itching, or a tingling sensation. The symptoms could appear long before the rash comes out by several days. Others might fall into this category and may experience headaches, photophobia (light sensitivity), and malaise in the initial stage of the disease [2].

Although the causes that make the VZV reactivate and bring about the HZ are not yet fully clear. For the individuals, their risk for HZ will rise when their cell-mediated immunity to VZV becomes weak. Aging may result in the weakening of an individual’s immune system and also from illnesses and medications that have suppressive effects on immunity, such as cancer, especially leukemia and lymphoma, human immunodeficiency virus (HIV), and taking immunosuppressive medications, including steroids and chemotherapy [3].

HZ risk is 50% lifetime in people, who are over 80 years old but live. The US statistics claim that 2.6 billion dollars are spent on direct medical costs per annum due to HZ [4]. In China, this employment issue might be estimated to cost the economy directly 216 million dollars each year. Statistics of world burden vary widely due to different epidemiological data availability systems in different countries [5]. Postherpetic neuralgia (PHN) is the most frequently observed sequel of HZ. PHN is pain that lingers in the region where the rash once stood for more than 90 days after the onset of the rash. PHN can persist over weeks or months, and sometimes - and that is not a trivial matter - for years [2].

The chance that a person would have PHN after HZ is higher when she is older. Older adults experience pain more frequently and in a more chronic fashion than younger people. It has been estimated that about 9% to 14% of people the age of 60 and older with HZ will have PHN. PdN is rare under 40 years of age [6]. According to the Centers for Disease Control and Prevention (CDC), the Shingrix (live attenuated zoster vaccine, or RZV) is recommended against shingles and other related complications. The CDC suggests that the recombinant vaccine should be given in two doses separated by 2 to 6 months to adults aged 50 years and above or adults aged 19 years and above who have or would have immunodeficiency or immune suppression due to disease or therapy [2].

In Saudi Arabia, the Ministry of Health advises vaccination for adults older than 50 who are immune competent as well as immunocompromised adults aged eighteen and older. The vaccine is administered in a similar way as the two-stroke, two to six-month interval. In the eastern part of Saudi Arabia, a 2023 research study that focused on the evaluation of HZ vaccination knowledge, practice, and attitude among the general population, found that about 75% of participants are willing to have the vaccine when their doctor recommends it to them [7].

In the UAE, a study published in 2022 to assess the knowledge, attitudes, and practices of the population towards HZ vaccination, revealed that participants' knowledge of the HZ vaccine was inadequate, and healthcare providers should encourage people aged 50 years old to take it [8]. The Zoster recombinant vaccine for adults is one of the most recent additions to the preventive measures provided in PHCs. The most common access route to this measure is through PHCs and patients are encouraged to direct their inquiries towards the primary care physician. While general practitioners in PHCs are provided educational material on the vaccine, most residents are not [9]. This creates variety in knowledge and attitudes among family residents based on their individual research and knowledge on the topic, affecting their ability to provide education to their patients. Another study suggests that educational projects on HZ and its vaccine which would mainly cover high-risk groups should be launched to render the population knowledgeable. Moreover, the role of healthcare workers' recommendation of the HZ vaccine to the eligible population should be emphasized, as it is one of the critical determinants of vaccine uptake [10].

The main purpose of the study is to evaluate the knowledge, behaviors, and attitudes of residents in Riyadh, Kingdom of Saudi Arabia, of family doctor residency programs toward the shingles vaccines. For instance, the research would want to establish the level of familiarity of the community with the recommendations for shingles vaccination as well as their current habits of recommending and administering the vaccine to the aged patients who qualify. Moreover, the research aims to find out any existing perceived barriers and obstacles that the residents of the facility endure while encouraging and implementing shingles immunization in their professional practice. The study will attempt to disclose these factors in order to provide the necessary information that can be used to come up with policies that will champion shingles vaccination among the targeted population.

## Materials and methods

The research was conducted at the residency program facilities for family medicine, which is located in Riyadh, the capital of Saudi Arabia, including institutions such as King Fahad Medical City (KFMC), King Saud Medical City (KSMC), and others. Inclusion criteria included the residents from the programs who agreed to participate were included in the study. Exclusion criteria included the residents not actively involved in residency training due to program freeze, leave of absence, or dropout were excluded. This ensured the study focused on currently engaged residents.

The sample size used in this study was N=154. Residents in the graphical levels (R1, R2, R3) were invited to participate. The data were collected using paper questionnaires, and the KFMC Institutional Review Board provided ethical approval. The study population includes residents at all levels (R1, R2, R3) who are under family medicine residency programs within different institutions in Riyadh, Saudi Arabia. The residents were investigated with their details being obtained to establish their understanding of the shingles vaccine and its practices and attitudes.

The research was carried out at several sites of residency programs for family medicine in Riyadh, Saudi Arabia. These institutions were KFMC, KSMC, King Abdulaziz Medical City (KAMC), King Faisal Specialist Hospital and Research Center (KFSHRC), King Saud University Medical City (KSUMC), Prince Sultan Military Medical City (PSMMC), Security Forces Hospital (SFH), and King Abdullah bin Abdulaziz University Hospital. This distribution of institutions across Riyadh means that the family medicine residents coming in are from different settings.

The participation of various institutions from across Riyadh reduces the specificity of the study findings. The study recruits a large population coverage of family medicine residents within the region, with different socio-economic characteristics and system settings. Such diversity assists in coming up with generalizations that are likely to be representative of the entire population of the family medicine residents in Riyadh, thus offering results that are likely to be replicable in other similar urban settings in Saudi Arabia.

The study uses a cross-sectional design for examining the knowledge, practice, and attitude of residents in family medicine residency programs in Riyadh City, Saudi Arabia, toward the shingles vaccine. This design enables gathering data at a one-time point, which represents a picture of the summation of the circumstances of the residents of different levels of the program [11]. The study will carry out a survey of residents from various institutions at the same time in order to deeply understand the subject matter that is relevant to the population within the specified area. The survey was administered to participants in person at the headquarters of family medicine residency programs in Riyadh, Saudi Arabia. Researchers personally invited residents from all levels (R1, R2, R3) to participate and explain the objectives of the study. Participants then completed the survey on paper questionnaires provided by the researchers.

The data collection tool employed in this study was a structured questionnaire divided into sections to cover all the aspects of knowledge, attitude, and practices as they relate to the recombinant zoster vaccine. The questionnaire was divided into four main sections: participants’ demographic profiles, their perception of the zoster vaccine, their attitudes toward the vaccine, and their practices in the management of vaccination. The sections included about 10-15 questions each; the questions were formulated in a way that would allow obtaining detailed, specific information in view of the study objectives. The questions were derived from the literature review, local public health data, and interviews with experts to make sure that issues under consideration are not missed. The questionnaire was produced prior to the administration and was pre-tested on a sample of the residents to make sure that all questions were comprehensible and none of the questions are sensitive. From the information received from the pilot test, slight changes were made to the questions to increase their clarity and efficiency. To ensure confidentiality, the questionnaires did not include any identifying information. Researchers were available on-site to address any questions or concerns raised by participants during the survey administration process.

A series of tests were implemented using statistical assertions. Key metrics such as mean, median, mode, standard deviation, and variance for each variable (gender, age, level of education or training, program enrollment, and type of VZV were covered). For example, it was ascertained that variations calculated to the means corresponded to the reported values up to a certain level of tolerance and that medians were accurate in pointing at the center of distributions. To confirm the validity of modes, the obtained data were checked to match the most commonly used responses, whereas standard deviations were calculated to determine the variability of the data. The variance was computed to come up with the variability within each of the variables. In total, these tests sought to validate the quality of the descriptive statistics provided, and their replicability for the next analyses and conclusions.

The approach used in this study drew from some principles from a related study done in the UAE on the vaccination for HZ [8]. The UAE study also revealed a number of knowledge deficits and misconceptions regarding HZ and its vaccine, thus the need for extensive educational initiatives. It also highlighted an important source of information which is the healthcare providers as far as the decision to vaccinate or not was concerned. These findings resulted in the addition of specific questions about sources of vaccine information, misconceptions, and the beliefs and behaviors of the family medicine residents to define targets for intervention to enhance the vaccination status of healthcare workers.

## Results

The information received is useful in terms of understanding the respondents’ characteristics, their perceptions of vaccination, as well as their knowledge of the process in general. Most of the respondents are male, within the age range of 25-30 years, and from the KFMC-MOH Cluster 2 Program. In regard to the vaccine, there is a prevalent utilization of the live attenuated VZV among the respondents. Concerning the perceptions and the prescription, there is a trend to prescribe the VZV in most of the adult groups such as the medically-free adults in the age of 18-49, immunocompromised persons as well as healthy adults who are above 50 years of age. The majority of the respondents indicate that they have the vaccine available in their place of practice, which enhances vaccine availability. However, the findings relating to the knowledge of timings and guidelines of vaccination show misguided and uncertain responses. A considerable percentage of respondents provide wrong information about the correct interval between doses, which is two to four weeks, and many are not aware of the requirement to repeat the vaccine series if the mentioned interval is violated. Moreover, controversies exist about the role of the vaccine for patients who have had shingles and the requirement of prior chickenpox infection or a positive VZV serology in the vaccine administration. These findings underscore the importance of increasing the public’s awareness and improving the directions provided to healthcare personnel to ensure they have accurate and stable perceptions about the use of vaccines. It is therefore important to fill these gaps to enhance the practices of vaccination and the achievement of proper immunization.

Demographics and general information

The demographic characteristics and general information about the respondents are given in Table 1. These are gender, age, level of education or training, the program the respondents are in, and the available VZV vaccine. Table 1 gives the mean, median, mode, standard deviation, and variance for each of the variables giving a broad picture of the distribution of the data. For instance, the mean age is 1.97 with the mode of 2, meaning that most of the respondents are in the second age group. Thus, the standard deviation and variance numbers give information on the dispersion within each category and differences in the characteristics of the respondents. This type of information is important for data analysis procedures for the sample and the general validity of further tests.

**Table 1:** Demographics and General Information (N=154)

Variable	Mean	Median	Mode	Std. Deviation	Variance
Gender	1.44	1.00	1	0.497	0.247
Age	1.97	2.00	2	0.342	0.117
Level	2.03	2.00	3	0.820	0.672
The program that you are in	3.66	2.00	1	2.694	7.260
The vaccine currently available for VZV is	1.26	1.00	1	0.440	0.194

Attitudes and recommendations

Table 2 shows the statistical characteristics of several recommendations and attitudes concerning vaccination for different groups of adults. Recommendations include the following variables: Medically healthy individuals between the ages of 18 and 49 years, immunocompromised persons between 18 and 49 years, healthy persons 50 years and above, and persons 50 years and above expecting or receiving immunosuppressive therapy. Also, it evaluates how available the vaccine is in the respondent’s places of practice. The mean, median, mode, standard deviation, and variance for each variable give an overview of general tendencies and fluctuations in the responses. For instance, the mean recommendation for the population that has no medical conditions and is between 18 and 49 years is 2.88, indicating a positive tendency of recommending the vaccine with a standard deviation of 0.843 suggesting moderate variation in the responses given by the respondents.

**Table 2:** Attitudes and recommendations (N=154)

Variable	Mean	Median	Mode	Std. deviation	Variance
Recommendation for medically-free adults aged 18-49	2.88	3.00	3	0.843	0.710
Recommendation for immunocompromised adults aged 18-49	2.94	3.00	3	0.798	0.636
Recommendation for healthy adults more than 50 years	2.84	3.00	2	0.801	0.642
Recommendation for adults more than 50 anticipating bone marrow or organ transplant not yet on immunosuppressive	2.50	2.50	2	1.116	1.245
Recommendation for adults more than 50 receiving immunosuppressive therapy	2.51	2.50	4	1.127	1.271
Is the vaccine available in your place of practice	1.06	1.00	1	0.356	0.126

Vaccination timings, recommendations, and information sources

Table 3 illustrates the statistical measures for various vaccination timing recommendations, beliefs, and sources of immunization information. It includes data on appropriate intervals between vaccine doses, actions if the recommended interval is exceeded, and recommendations for specific patient groups such as those who have had shingles or are on low-dose methotrexate. The table also covers opinions on the necessity of chickenpox history for vaccination and the handling of side effects. Additionally, it highlights healthcare providers' preferences for referring immunocompromised patients to specialists and their comfort in making decisions for such patients. The mean values, often close to 1, suggest general agreement or affirmation, with standard deviations indicating variability in opinions. Higher mean values for variables like preference to refer immunocompromised patients (3.42) and comfort in decision-making (3.31) suggest more diverse views. Information sources such as journal articles, conferences, and the internet are also evaluated, with mean values around 2.5, indicating moderate reliance on these sources for immunization information.

**Table 3:** Knowledge and practices (N=154) VZV: Varicella Zoster Vaccine

Variable	Mean	Median	Mode	Std. deviation	Variance
Time between doses should be 1-2 months	1.74	1.00	1	0.892	0.795
Time between doses should be 2-6 months	1.75	1.00	1	0.912	0.831
If recommended interval is exceeded repeat series	1.74	1.00	1	0.892	0.795
Get vaccine even if had shingles already	1.74	1.00	1	0.892	0.795
Wait at least 5 years if already had live attenuated shingles vaccine	1.74	1.00	1	0.892	0.795
Any adult patient on low dose methotrexate should get vaccine	1.78	1.00	1	0.916	0.840
History of chickenpox or positive VZV serology needed to get vaccine	1.83	1.00	1	0.927	0.860
If side effects affect normal activities should not receive second dose	1.80	1.00	1	0.917	0.842
Prefer to refer immunocompromised patients to specialist	3.42	3.00	1	1.337	1.788
Comfortable making decision on immunocompromised patients	3.31	3.00	5	1.335	1.782
Difficult to convince patients due to first dose side effects	3.03	3.00	3	1.252	1.568
Get immunization information from journal articles	2.51	2.50	2	1.074	1.154
Get immunization information from conferences	2.50	2.50	2	1.068	1.141
Get immunization information from internet sources	2.51	2.50	2a	1.074	1.154
Get immunization information from internet aggregation sources	2.51	2.50	2	1.080	1.166

Demographics and vaccine attitudes

Table 4 provides a comprehensive breakdown of the demographic characteristics of the respondents, their attitudes toward vaccine recommendations, and their sources of immunization information. It shows that the majority of respondents are male 87 (56.5%) and aged 25-30 years 136 (88.3%). Most are in the R3 level of training 54 (35.1%) and primarily come from the KFMC-MOH Cluster 2 program 61 (39.6%). The prevalent VZV available is the live attenuated vaccine 114 (74.0%). Recommendations for various groups, such as medically free adults aged 18-49 and immunocompromised adults, are detailed, with a significant portion recommending or strongly recommending the vaccine. The table also reveals that most respondents have the vaccine available at their place of practice 149 (96.8%) and encounter varying degrees of patient refusal, often due to misconceptions about risk and fear of side effects. Additionally, it highlights the reliance on journal articles 44 (28.6%), conferences 45 (29.2%), and internet sources 45 (29.2%) for immunization information, reflecting diverse informational resources among healthcare professionals.

**Table 4:** Frequencies and percentages of variables (N=154) VZV: Varicella-Zoster Vaccine

Variable	Category	Frequency (%)
Gender	Male	87 (56.5)
	Female	67 (43.5)
Age	<25 years old	11 (7.1)
	25-30 years old	136 (88.3)
	30 years old	7 (4.5)
Level	R1	49 (31.8)
	R2	51 (33.1)
	R3	54 (35.1)
The program that you are in	KFMC–MOH Cluster 2	61 (39.6)
	KAMC	18 (11.7)
	KFSHRC	3 (1.9)
	KSMC–MOH Cluster 1	2 (1.3)
	KSUMC	20 (13.0)
	PSMMC	21 (13.6)
	SFH	9 (5.8)
	King Abdullah bin Abdulaziz University Hospital	20 (13.0)
The vaccine currently available for Varicella Zoster vaccine is	Live attenuated vaccine	114 (74.0)
	Recombinant inactive vaccine	40 (26.0)
Recommendation for medically free adults aged 18-49	NO recommendations against or for	5 (3.2)
	Don’t recommend	50 (32.5)
	Recommend the vaccine	58 (37.7)
	Strongly recommend the vaccine	41 (26.6)
Recommendation for immunocompromised adults aged 18-49	NO recommendations against or for	4 (2.6)
	Don’t recommend	42 (27.3)
	Recommend the vaccine	68 (44.2)
	Strongly recommend the vaccine	40 (26.0)
Recommendation for healthy adults more than 50 years	NO recommendations against or for	1 (0.6)
	Don’t recommend	60 (39.0)
	Recommend the vaccine	55 (35.7)
	Strongly recommend the vaccine	38 (24.7)
Recommendation for adults more than 50 anticipating bone marrow or organ transplant not yet on immunosuppressive therapy	NO recommendations against or for	38 (24.7)
	Don’t recommend	39 (25.3)
	Recommend the vaccine	39 (25.3)
	Strongly recommend the vaccine	38 (24.7)
Recommendation for adults more than 50 receiving immunosuppressive therapy	NO recommendations against or for	38 (24.7)
	Don’t recommend	39 (25.3)
	Recommend the vaccine	37 (24.0)
	Strongly recommend the vaccine	40 (26.0)
Is the vaccine available in your place of practice	Yes	149 (96.8)
	Unsure	5 (3.2)
How often do your patients refuse the vaccine when you recommend it for them	Never	38 (24.7)
	Rarely	39 (25.3)
	Sometimes	39 (25.3)
	Always	38 (24.7)
Reason for refusal: thinks they are not at risk of developing shingles	Never	38 (24.7)
	Rarely	39 (25.3)
	Sometimes	40 (26.0)
	Always	37 (24.0)
Reason for refusal: does not think they ever had chickenpox	Never	34 (22.1)
	Rarely	39 (25.3)
	Sometimes	40 (26.0)
	Always	41 (26.6)
Reason for refusal: fear of immediate side effects	Never	35 (22.7)
	Rarely	41 (26.6)
	Sometimes	42 (27.3)
	Always	36 (23.4)
Reason for refusal: fear of long-term side effects	Never	35 (22.7)
	Rarely	40 (26.0)
	Sometimes	42 (27.3)
	Always	37 (24.0)
Reason for refusal: thinks shingles are not severe	Never	86 (55.8)
	Rarely	22 (14.3)
	Sometimes	46 (29.9)
Time between doses should be 1-2 months	False	88 (57.1)
	True	17 (11.0)
	I don't know	49 (31.8)
Time between doses should be 2-6 months	False	86 (55.8)
	True	22 (14.3)
	I don't know	46 (29.9)
If recommended interval is exceeded repeat series	False	86 (55.8)
	True	22 (14.3)
	I don't know	46 (29.9)
Get vaccine even if had shingles already	False	86 (55.8)
	True	22 (14.3)
	I don't know	46 (29.9)
Wait at least 5 years if already had live attenuated shingles vaccine	False	85 (55.2)
	True	18 (11.7)
	I don't know	51 (33.1)
Any adult patient on low dose methotrexate should get vaccine	False	81 (52.6)
	True	18 (11.7)
	I don't know	55 (35.7)
History of chickenpox or positive VZV serology needed to get vaccine	False	83 (53.9)
	True	19 (12.3)
	I don't know	52 (33.8)
If side effects affect normal activities should not receive second dose	False	90 (58.4)
	True	12 (7.8)
	I don't know	52 (33.8)
Prefer to refer immunocompromised patients to specialist	Strongly agree	14 (9.1)
	Agree	24 (15.6)
	Neutral	51 (33.1)
	Disagree	13 (8.4)
	Strongly disagree	52 (33.8)
Comfortable making decision on immunocompromised patients	Strongly agree	17 (11.0)
	Agree	24 (15.6)
	Neutral	53 (34.4)
	Disagree	15 (9.7)
	Strongly disagree	45 (29.2)
Difficult to convince patients due to first dose side effects	Strongly agree	20 (13.0)
	Agree	33 (21.4)
	Neutral	49 (31.8)
	Disagree	27 (17.5)
	Strongly disagree	25 (16.2)
Get immunization information from journal articles	Never	23 (14.9)
	Rarely	47 (30.5)
	Sometimes	40 (26.0)
	Always	44 (28.6)
Get immunization information from conferences	Never	21 (13.6)
	Rarely	48 (31.2)
	Sometimes	40 (26.0)
	Always	45 (29.2)
Get immunization information from internet sources	Never	23 (14.9)
	Rarely	46 (29.9)
	Sometimes	40 (26.0)
	Always	45 (29.2)
Get immunization information from internet aggregation sources	Never	23 (14.9)
	Rarely	47 (30.5)
	Sometimes	40 (26.0)
	Always	44 (28.6)

Vaccination knowledge

**Figure 1:**
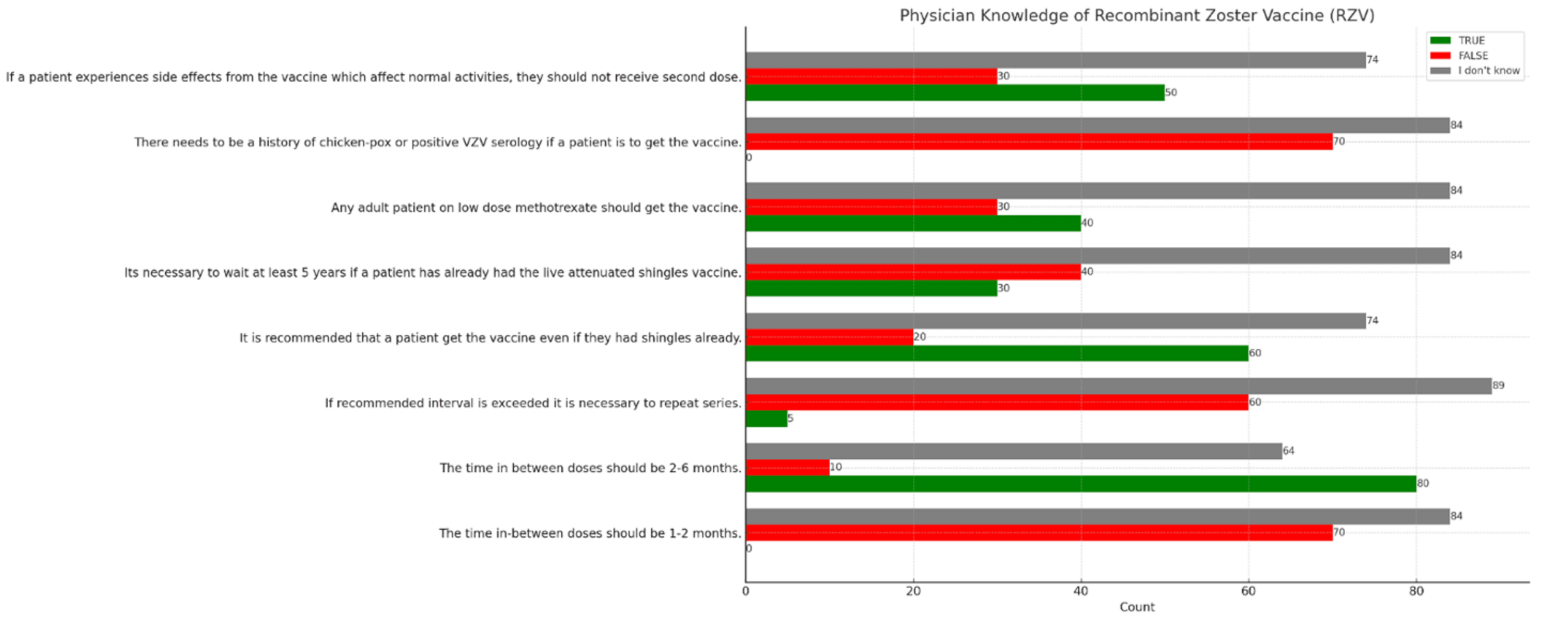
Vaccination knowledge among respondents (N=154) VZV: Varicella-Zoster Vaccine

Table 5 highlights the knowledge and misconceptions among respondents regarding the VZV guidelines. For example, a significant number of respondents incorrectly believe that the time between doses should be one to two months, with 84 respondents unsure. Conversely, there is better knowledge regarding the two to six-month interval, with 80 respondents answering correctly. Similarly, many respondents are uncertain about the need to repeat the series if the recommended interval is exceeded and whether patients should receive the vaccine if they had shingles before. These responses indicate areas where further education and clarification are needed to ensure healthcare professionals have accurate information regarding vaccine administration (Figure 1).

**Table 5:** Vaccination knowledge (N=154) VZV: Varicella-Zoster Vaccine

Question	True	False	I don’t know	Correct answer
The time in-between doses should be 1-2 months	0	70	84	False
The time in between doses should be 2-6 months	80	10	64	True
If recommended interval is exceeded it is necessary to repeat series	5	60	89	False
It is recommended that a patient get the vaccine even if they had shingles already	60	20	74	True
Its necessary to wait at least 5 years if a patient has already had the live attenuated shingles vaccine	30	40	84	False
Any adult patient on low dose methotrexate should get the vaccine	40	30	84	False
There needs to be a history of chicken-pox or positive VZV serology if a patient is to get the vaccine	0	70	84	False
If a patient experiences side effects from the vaccine which affect normal activities, they should not receive second dose	50	30	74	False

## Discussion

Demographics and general information

The current study provides a detailed demographic profile of respondents, including gender, age, level of education or training, program enrollment, and type of VZV vaccine available. Statistical measures such as mean, median, mode, standard deviation, and variance are reported for each variable. For instance, the mean age is 1.97 with a mode of 2, indicating that the majority of respondents fall into the second age category. The gender distribution shows a mean of 1.44, suggesting a slightly higher proportion of one gender, with a standard deviation of 0.497 indicating moderate variability. These statistics provide a foundational understanding of the sample population and their characteristics. Grabenstein et al. conducted a study on the demographic, clinical, and attitudinal characteristics of people vaccinated by pharmacists across multiple states. The average age of respondents was 54 ± 15 years, with 60% being women. Nearly a quarter of respondents were aged 65 years or older, and about 9% were younger than 65 but took medication for chronic conditions such as heart or lung disease or diabetes. This contrasts with the current study, where the mean age is significantly lower. Grabenstein et al. emphasized the satisfaction of respondents with pharmacist-administered vaccinations, citing access, proximity, trust, convenience, and cost as major advantages [12]. Sukumaran et al. examined the demographic characteristics of the vaccine safety datalink (VSD) population, comparing them to the general US population. The VSD data included over eight million individuals, representing a broad range of demographic groups. The study found no major differences in sex, race, ethnicity, or educational attainment between the VSD and the US population, although middle-income populations were comparable, and lower-income populations were slightly underrepresented in the VSD. This aligns with the comprehensive demographic data provided in the current study, which also aims to ensure that various demographic groups are adequately represented [13].

Attitudes and recommendations

The current study focuses on the perception and suggestions on vaccination for other categories of adults apart from those eligible for COVID-19 vaccines such as medically free adults ranging from 18 to 49 years, immunocompromised adults ranging from 18 to 49 years, healthy adults with 50 years and more, and adults with 50 years and more who expect to receive or are planning for immunosuppressive therapy. The descriptive statistics which include Mean, Median, Mode, standard deviation, and variance give an outlook of the general trends and fluctuations in the response. For instance, the mean recommendation for the medically insured population of people between 18 and 49 years is 2.88, and the standard deviation of 0.843, which shows that it has moderate fluctuation in the responses given to the questions. The data are used in the formulation of the healthcare professionals’ recommendations and any possible requirements for further details or care. Luyten et al. used the modified VHS to determine the vaccine hesitancy level among the UK population. They were able to conclude that hesitancy was significantly related to the respondent’s age, gender, and education level. They identified two main subscales within the VHS: the social factor could be due to a lack of confidence especially on the need to take vaccines and fear of side effects. This study also observes that there is a large part of the population with a middle attitude toward vaccination; however, these attitudes do not correlate with ordinary sociodemographic factors; therefore, the population’s attitudes toward vaccinations can vary depending on demographic factors [14]. Esteban-Vasallo et al. conducted a population survey in Spain to evaluate the level of information of healthcare workers about vaccines. Specifically, when it comes to knowledge about vaccines 7% of the respondents said that they did not receive enough information. This perception was associated with lower vaccination with the influenza vaccine and a higher degree of skepticism about the use of vaccines. Specifically, female gender, lower household income, and poor self-perceived health were found to be statistically related to the perception of insufficient information. This corresponds with the research questions of the current study, which investigates the sources of immunization information and emphasizes the significance of accurate and comprehensive vaccine information [15].

Scheduling of vaccines, advisory, and information retrieval

The current study depicts the average, standard deviation, skewness, and kurtosis of different suggestions and beliefs on vaccination timing and the sources of immunization information. The data embraces the proper time between the administration of vaccines, what is to be done in the situation when the time difference is crossed, and the guidelines for the particular patients. The mean values, that are near 1, indicate agreement or affirmation while standard deviations denote variation in opinions. As for the mean values, they are higher for such variables as preference to refer to immunocompromised patients (3.42) and comfort in decision-making (3.31), which indicates more diverse opinions. Other information types like journals, conferences, and internet sources are also assessed and they show a moderate level of dependence, on these sources regarding immunization information. Schwarzinger et al. investigate COVID-19 vaccine reluctance in a sample of working-age people in France. The discrete choice experiment conducted by their study aimed at determining the effect of vaccine attributes such as the effectiveness of the vaccine, potential side effects, and the manufacturing company. This paper highlighted the degree to which vaccine acceptance varied by these attributes and stressed the need for unambiguous information and appropriate interventions to counteract vaccine reluctance. This stresses knowledge by health care providers and the relevance of the multiple sources of information for the decisions, as pointed out in the current study [16]. Wang et al. used a study that took place in Hong Kong to identify the factors, which influence COVID-19 vaccination acceptance. This saw that the acceptance rate was dependent on the effectiveness of the vaccine, safety, and the involvement of the general practitioners. The study also established that exposure to vaccination information on social media was very high among the respondents and that was a very strong significant factor explaining vaccine refusal. This study connects to the concern for information, and people leaning on the healthcare professionals for information when it comes to vaccines, which is in line with the present work’s emphasis on the availability of various information sources [17].

Demographics and vaccine attitudes

In the present investigation, details on the demographic profile of the respondents, their perception of the vaccine recommendations, and information sources regarding immunization are presented. The study reveals that the larger portion of the users are male (56.5%) and within the age of 25-30 years (88.3%). The majority of them are in the R3 level of training and almost all of them were trained in the KFMC - MOH Cluster 2 program. The current type of VZV that is commonly used is the live attenuated vaccine according to 74.0%. There are recommendations presented for different populations, including people with no comorbidities aged 18-49 and immunocompromised individuals; a considerable number of them are advised to get the vaccine or even strongly recommended to do so. Furthermore, the survey establishes that most participants have the vaccine at the workplace (96.8%) and experience different levels of refusal from the patients stemming from misconceptions regarding risk and perceived side effects of the vaccine. Taylor et al in 2011 sought to establish HPV vaccine coverage in the United States and they noted that while the initiation of the vaccine was age-dependent it was not by race or poverty level. It also reveals that to a great extent, 19-26-year-olds with private insurance in the United States started vaccination more than those with public insurance. This is in line with the present study’s investigation of the demographic factors toward vaccine acceptance and the role of availability and insurance in vaccination. Jantzen et al. looked at the socio-demographics of COVID-19 vaccine hesitancy in Quebec and identified household income and place of birth as predictors of hesitancy. This study therefore supports the call for public health interventions to target socio-economic factors and tailor its messages in a bid to eliminate vaccine skepticism as revealed in the current study on the various factors that shape one’s perception of vaccines [18].

Knowledge and practices

The current study aims at establishing the level of awareness and the level of ignorance that respondents have in so far as VZV guidelines are concerned. For instance, a large proportion of the sampled participants give the wrong information regarding the time gap between doses as one to two months with other aspects of vaccine administration being unclear. Such responses show where there is a need to educate and make clarifications so that those in the healthcare practice have the right information. Raheja et al. on willingness of the older adults to participate in vaccine-related clinical trials identified that male participants were more willing than female participants. The majority of the participants were willing to travel up to 25 miles to a research clinic and a large number of them were not willing to participate if the trial outcomes were not disclosed. These findings stress the need to increase public participation in clinical trials focusing on vaccines, which in the present research can be associated with the role of enhancing communication and knowledge in the given field [19]. Chao et al. compared the characteristics of patients who decided to start the HPV vaccine with those who did not. This study showed that there is a correlation between the rates of HPV vaccine uptake and some variables like higher income per neighborhood, Physician office visits, and history of previous vaccination against influenza [20]. These findings are useful for making sense of the findings in observational safety studies and generating ideas for designing population-specific HPV vaccination programs, which is the emphasis of the current study, demographic factors’ impact on vaccine awareness and use.

Limitations

Some of the study limitations are response bias since all the data collected are self-reported, restricted generalizability of the study findings since the results are only applicable to the healthcare programs that were surveyed and recall bias that may influence the participants’ knowledge and attitudes toward VZV vaccination. Also, the study may not be representative of all regions or all types of healthcare settings, and therefore the generalizability of the findings may be compromised.

## Conclusions

This study underlines the variation in the advisories for vaccination among the healthcare workers, and the two different vaccines for VZV. The use of various information sources highlighted the importance of specific educational interventions to achieve the desired level of immunization consistency among healthcare facilities. Managing the vagueness and facilitating decision-making can positively impact vaccination rates and patient care in practice. It is essential to apply targeted education programs to make knowledge outcomes among residents equal; these programs should address the identified gaps in knowledge. Such programs can be in the form of workshops, seminars, and online modules specific to the level of residency training. Also, it is necessary to include constant and systematic evaluation and feedback procedures to guarantee that the educational interventions succeed and meet the changing needs of the residents.

The results of this study suggest that future research should employ longitudinal study designs in order to investigate shifts in knowledge, attitudes, and practices over time. Furthermore, enlarging the investigation to other areas of the Kingdom of Saudi Arabia can give a wider picture of the national situation concerning zoster vaccination. Using other qualitative approaches like interviews and focus group discussions could also provide a more profound understanding of the challenges as well as the enablers of vaccination among healthcare workers.

## Appendices

Questionnaire used in the study 

Practices, Attitudes, and Knowledge Regarding Recombinant Zoster Vaccine among Family Medicine residents in Riyadh, Saudi Arabia

You are formally invited to participate in This questionnaire that is designed to assess the family medicine resident’s knowledge, attitudes and practices regarding the varicella zoster recombinant vaccine. 

If you agree to participate, your participation will involve completing a survey.

It should take no more than 5-7 minutes. Your name will not appear on your completed survey, and no identifying information is being collected as part of this survey.

By participating in the survey, you are giving permission for the investigator to use your information for research purposes

Thank you

Gender

o   Male

o   Female

Age

o   <25 years old

o   25-30 years old

o   > 30 years old

Level

o   R1

o   R2

o   R3

The program that you are in :-

o   King Fahad Medical City (KFMC - MOH Cluster 2)

o   King Saud Medical City (KSMC - MOH Cluster 1)

o   King Abdulaziz Medical City at National Guard (KAMC)

o   King Faisal Specialist Hospital and Research Center (KFSHRC)

o   Security Forces Hospital (SFH)

o   King Saud University Medical City (KSUMC)

o   Prince Sultan Military Medical City (PSMMC)

o   King Abdullah bin Abdulaziz University Hospital

Knowledge

**Table 6:** Questionnaire for sections (knowledge, practices, barriers, and attitudes)

	Live attenuated vaccine	Recombinant inactive vaccine
The vaccine currently available for Varicella Zoster vaccine is		

	Strongly recommend the vaccine	Recommend the vaccine		No recommendation against or for		Don’t recommend			Strongly don’t recommend
a. What is your recommendation for each patient type Medically-free Adults aged 18-49									
b.immunocompromised adults aged 18-49									
c. Healthy adults more than 50 years									
d. Adults more 50 years old anticipating bone marrow or organ transplant not yet on immunosuppressive therapy									
e. Adults more than 50 years old receiving immunosuppressive therapy									
f. Adults more than 50 on low dose methotrexate									
g. Adults more than 50 receiving an immunomodulator									
h. Adults more than 50 with HIV with CD-4 count more than 200									
i. Adults more than 50 on chemotherapy									
			Yes		No			Unsure	
Is the Vaccine available in your place of practice									
			Multiple days		Multiple weeks			Months	
If answered no How long has it been out of stock									

	Less than 5%	5-25%	More than 25%	Unsure
How often does your patients refuse the vaccine when the recommendations for it based on the guidelines				

	True	False	I don’t know
a. The time in-between doses should be 1-2 months			
b. The time in between doses should be 2-6 months			
c. If recommended interval is exceeded it is necessary to repeat series			
d. It is recommended that a patient get the vaccine even if they had shingles already			
e. Its necessary to wait at least 5 years if a patient has already had the live attenuated shingles vaccine			
f. Any adult patient on low dose methotrexate should get the vaccine			
g. There needs to be a history of chicken-pox or positive VZV serology if a patient is to get the vaccine			
h. If a patient experiences side effects from the vaccine which affect normal activities, they should not receive second dose			

	Strongly agree	Agree	Disagree	Strongly Disagree
a. I prefer to refer immunocompromised patients to a specialist to make the decision on the vaccine				
b. I am comfortable making my decision on immunocompromised patients regarding the vaccine				
c. Due to the side effects of the first dose side effects, I find it difficult to convince patients to get the vaccine				

	Major barrier	Moderate barrier	Minor barrier	Not a barrier
a. Patients are unwilling to go to other center to get the shingles vaccine				
b. There are limited supply of vaccine				
c. I forget to discuss the vaccine with my patients				
d. I have concerns about the safety of the vaccine				
e. The patients have concerns regarding the safety of the vaccine				
f. I have concerns regarding effectiveness of the vaccine				
g. I believe there’s insufficient information about the vaccine				
h. I have concerns regarding length of protection afforded by the vaccine				
i. I don’t think my patients are at risk of getting postherpetic neuralgia				
j. More pressing medical issues take precedence				
k. The need for 2 doses is a barrier to administration				
l. I believe the vaccination recommendations will change soon				

	Always	sometimes	rarely	never
a. Patient thinks they are not at risk of developing shingles				
b. Patient does not think they ever had chicken-pox				
c. Fear of immediate side effects				
d. Fear of long-term side effects				
e. Patient doesn’t think shingles is a severe disease				

	Always	sometimes	rarely	never
a. Journal articles				
b. Conferences				
c. Internet sources, such as webcasts, online interactive modules, recorded presentations				
d. Internet aggregation sources, such uptodate, Medscape, AAFP				
e. Ministry of health issued guidance documents				
